# Accuracy of Diagnostic Biopsy for Cutaneous Melanoma: Implications for Surgical Oncologists

**DOI:** 10.1155/2013/196493

**Published:** 2013-09-11

**Authors:** Tina J. Hieken, Roberto Hernández-Irizarry, Julia M. Boll, Jamie E. Jones Coleman

**Affiliations:** ^1^Department of Surgery, NorthShore University HealthSystem, Skokie Hospital, Skokie, IL, USA; ^2^Rush University Medical Center, Chicago, IL, USA; ^3^Rush Medical College, Chicago, IL, USA; ^4^Department of Surgery, Mayo Clinic, 200 First Street SW, Rochester, MN, USA

## Abstract

*Background and Objectives*. While excisional biopsy is recommended to diagnose cutaneous melanoma, various biopsy techniques are used in practice. We undertook this study to identify how frequently final tumor stage and treatment recommendations changed from diagnostic biopsy to final histopathology after wide local excision (WLE). *Methods*. We compared the histopathology of the dermatopathologist-reviewed diagnostic biopsy and final WLE in 332 cutaneous melanoma patients. *Results*. Tumor sites were extremity (51%), trunk (33%), and head/neck (16%). Initial biopsy types were excisional (56%), punch (21%), shave (18%), and incisional (5%). Most diagnostic biopsies were margin positive regardless of technique, and 36% of patients had residual melanoma on WLE. T-stage changed in 8% of patients, of whom 59% were diagnosed by punch biopsy, 15% by incisional biopsy, 15% by shave biopsy, and 11% by excisional biopsy (*P* < 0.0001). Treatment recommendations changed in 6%: 2% after excisional biopsy, 5% after shave biopsy, 18% after punch biopsy, and 18% after incisional biopsy (*P* < 0.0001). *Conclusions*. Although most biopsy margins were positive, T-stage and treatment changed for only a minority of melanoma patients. Our data provide valuable information to inform patient discussion regarding the likelihood of a change in prognosis and the need for secondary procedures after WLE. These data support the superiority of dermatopathologist-reviewed excisional biopsy when feasible.

## 1. Introduction

The incidence of malignant melanoma continues to increase. It is estimated that more than 76,600 new cases of melanoma will be diagnosed in the United States in 2013, with 9,480 deaths attributed to this disease. The lifetime risk for the development of melanoma is now 1 in 35 for males and 1 in 54 for females [1]. The thickness of the primary melanoma, as measured histopathologically, guides treatment and provides important prognostic and staging information; a proper dermatopathologist-reviewed diagnostic biopsy is essential for appropriate management of the newly diagnosed melanoma patient. Currently, excisional biopsy is the recommended diagnostic procedure for melanoma [2–5]. However, in practice, cutaneous melanoma is diagnosed by a variety of biopsy techniques, and the proportion of cutaneous melanomas diagnosed by nonexcisional biopsy techniques is increasing [6].

Studies suggest that dedicated dermatopathology review of a pigmented lesion biopsy is important to establish a correct diagnosis [7, 8]. However, data is lacking on how the biopsy type, after dermatopathology review, correlates with final diagnosis and the need for secondary operations after planned therapeutic wide local excision (WLE). Extrapolation from prior studies is inadequate due to various factors including strict inclusion criteria, small study size, variable definitions of biopsy types and microstaging accuracy, comparisons made without dermatopathology review, and a lack of data on the effect of a stage change on treatment recommendations [8–15]. 

Ideally, preoperative consultation with the newly diagnosed melanoma patient includes a discussion of prognosis and treatment options. Both may change after definitive excision and histopathologic evaluation of the excised melanoma. Therefore, we undertook this study of our melanoma practice to identify how frequently the final tumor thickness, level, and T-stage changed after WLE, how this varied with biopsy type and margin status, and how often such changes resulted in altered treatment recommendations including the need for further surgery.

## 2. Materials and Methods 

After approval from our institutional review board, we identified 410 consecutive cutaneous melanoma patients entered prospectively into our Melanoma Registry at NorthShore University HealthSystem Skokie Hospital and without evidence of metastatic disease. Data were confirmed by review of pathology reports, operative notes and medical records. Forty-four patients were excluded due to incomplete data, and 34 patients were excluded from our cohort because they did not undergo WLE, leaving 332 patients with suitable and sufficient data for the analysis. All outside biopsies were reviewed by a dermatopathologist. The reviewed biopsy was used as the basis for comparison to the final WLE histopathology. Diagnostic biopsy types were classified as excisional, shave, punch, or incisional based upon the description of the procedure by the physician performing the biopsy. The majority of the shave biopsies were deep scoop shave biopsies. We defined the tumor thickness of the melanoma as the greater of either the biopsy or WLE specimen. Patients were staged using the AJCC 6th edition melanoma guidelines. Statistical analysis was performed using an SAS software package. The Chi-square or Fisher's exact test was used for analysis of categorical variables. One-way ANOVA, the Student's *t*-test (for normally distributed data), and the Mann-Whitney *U* test (for nonparametric data) were used for analysis of continuous variables. Mean values are reported as mean ± standard error of the mean. *P* values of <0.05 were considered significant.

## 3. Results

**Figure 1:**
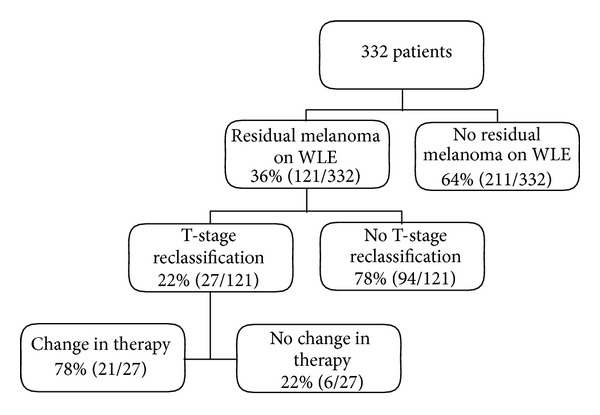
Effect of Finding Residual Melanoma on Wide Local Excision on T-Stage Change and Subsequent Change in Treatment.

Patient demographic and tumor data are summarized in Table 1. Diagnostic biopsy type was excisional in 187 patients (56%), punch in 68 patients (21%), shave in 60 patients (18%), and incisional in 17 patients (5%). Overall, 204 of 332 patients (61%) had a positive diagnostic biopsy margin. The likelihood of a positive biopsy margin and the degree or type of margin positivity (lateral and/or deep margin involvement) varied significantly by biopsy type as shown in Table 2. Residual melanoma was identified in the WLE specimen in 121 patients (36%), representing 11% of patients (14/128) with negative biopsy margins and 53% of patients (107/204) with positive biopsy margins (*P* < 0.0001). The likelihood of identifying residual melanoma in the WLE specimen according to diagnostic biopsy technique and diagnostic biopsy margin status is summarized in Tables 3 and 4. The effect of final histopathology at WLE on final staging and treatment recommendations is summarized in Figure 1. Among the 121 patients with residual melanoma identified in the WLE specimen, the Breslow depth was less than or equal to the biopsy depth in 68 cases (56%), while tumor thickness increased in 53 cases (44%) and tumor level increased in 26 cases (21%). T-stage reclassification occurred in 27 of 121 patients with residual melanoma in the WLE specimen (22%) or 8% of all cases. The likelihood of a change in tumor thickness, level, and stage also varied with biopsy type. Among the 27 cases in which T-stage changed after WLE, 16 patients (59%) were initially diagnosed by punch biopsy, 4 (15%) by incisional biopsy, 4 (15%) by shave biopsy, and 3 (11%) by excisional biopsy. Forty-six of 332 melanomas (14%) were ulcerated, and in all cases this was noted on histopathology review of the biopsy; no additional cases of ulcerated melanoma were diagnosed after WLE. 

**Figure 2:**
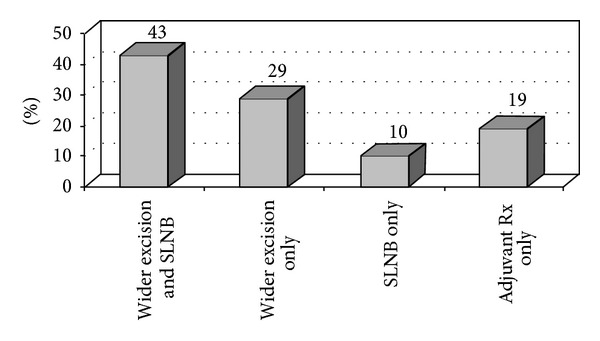
Changes in Treatment Recommendations After Wide Local Excision. Abbreviations: SLNB = sentinel lymph node biopsy; Rx = treatment.

Treatment recommendations were altered for 21 patients after WLE, representing 17% of patients with residual melanoma on WLE and 6% of the total patient population. The nature of these changes are shown in Figure 2. The need for additional treatment varied significantly with the type of biopsy performed: 3 of 187 excisional biopsy patients (2%), 3 of 60 shave biopsy patients (5%), 12 of 68 punch biopsy patients (18%), and 3 of 17 incisional biopsy patients (18%) (*P* < 0.0001). Of these 21 patients, 15 (71%) required wider margins of excision, and 11 (52%) became candidates for sentinel lymph node biopsy. The details of these altered treatment recommendations are specified in Figure 2. An additional 18 patients with a positive sentinel node after sentinel lymph node biopsy were not considered as having a change in treatment recommendations for the purposes of this study. Variables associated with T-stage reclassification and modified treatment recommendations after WLE are shown in Table 5. Factors significantly associated with the likelihood of a change in T-stage included increasing melanoma diameter, increasing Breslow depth, punch or incisional biopsy technique, involved biopsy margins, and the type of positive biopsy margin. Twenty-four percent of patients diagnosed by punch or incisional biopsy, 12% of all patients with a positive biopsy margin, and 29% of patients with involvement of both deep and lateral biopsy margins were understaged by the biopsy. Alteration in treatment recommendations was associated with increasing melanoma diameter, punch or incisional biopsy technique, tumor-involved biopsy margins, and the type of positive biopsy margin. While 18% of patients diagnosed by punch or incisional biopsy required modification of the treatment plan after WLE, only 2% of patients diagnosed by excisional biopsy required additional treatment. Nine percent of all patients with a positive biopsy margin and 23% of patients with involvement of both deep and lateral biopsy margins required further treatment.

## 4. Discussion 

Accurate preoperative tumor staging is critical for determining appropriate treatment for melanoma patients including selection of appropriate resection margins, the need for sentinel lymph node biopsy, and consideration of adjuvant therapy. While the incidence of melanoma is increasing, the death rate from melanoma is increasing at a slower rate and even decreasing for some patient subgroups (i.e. younger patients and females) [16]. This improvement in survival is largely attributable to early diagnosis and prompt treatment. Patients with T1 melanomas have a 10-year melanoma-specific survival of >90% after surgical treatment alone, while those with thicker melanomas have a markedly poorer prognosis [17]. To diagnose melanoma at an early stage, excisional biopsy is recommended for evaluation of suspect pigmented lesions [2–5]. With increased awareness of melanoma and opportunities for screening, there has been a corresponding increase in the number of skin lesions biopsied. However, only 40–80% of melanomas are clinically suspected to be melanoma prior to biopsy [9, 15, 18]. 

Even guidelines that strongly encourage excisional biopsy of pigmented lesions state that incisional, punch, or shave biopsies may be appropriate in selected clinical circumstances. These include evaluation of large facial or acral lesions, lesion diameter >2 cm, concern about cosmesis or tissue laxity, or when the suspicion of melanoma is low [2, 4]. From a technical standpoint, a non-excisional biopsy is often used due to physician training and skills, time constraints, and available resources. The main hazards of partial biopsy are misdiagnosis, diagnostic uncertainty, and staging inaccuracy. The theory that incisional biopsy *per se* is harmful to melanoma patients and has a detrimental effect on prognosis has been refuted in several studies [19–22]. 

Consequently, the use of diagnostic techniques other than excisional biopsy is increasing, even for suspected melanomas [6]. The reported frequency of diagnostic excisional biopsy is variable, ranging from 10–86%, suggesting that in many cases, as in our series, melanoma is diagnosed by less than excisional biopsy, most often shave biopsy [7–9, 11, 13, 20, 23]. With this shift away from excisional biopsy for the diagnosis of melanoma, it is important for clinicians and patients to have data regarding the likelihood of a change in prognosis and treatment recommendations that may occur after WLE, despite expert review of the biopsy specimen.

In our study, most cutaneous melanoma patients were diagnosed by excisional biopsy, with the remainder having a shave, punch or incisional biopsy. Regardless of the technique, most diagnostic biopsies had positive margins, and many had residual melanoma on WLE. We noted a correlation with biopsy type favoring excisional biopsy, followed by shave biopsy (mainly deep scoop shaves). However, treatment recommendations were modified for only a small percentage, ranging from 2% to 18% depending upon biopsy technique, in our series in which all diagnostic biopsies were dermatopathologist reviewed prior to definitive operation. 

Several investigators have suggested that it is best to diagnose melanoma in a patient with a suspicious lesion by whatever means readily available to the physician who first evaluates the patient [14, 24, 25]. They assert that it is better to use non-excisional biopsy techniques to make a tissue diagnosis than to do no biopsy at all or to ablate the lesion, and that a poorly planned or performed excisional biopsy may be detrimental. Others suggest that not only do partial biopsies of pigmented lesions hold an increased risk of microstaging inaccuracy when a diagnosis of melanoma is made but also caution that partial biopsies are highly associated with misdiagnosis and malpractice lawsuits [4, 8, 23]. Potential problems with partial biopsy include sampling error and an inability to evaluate the architecture of the intact lesion. While guidelines suggest that the thickest, darkest, or most recently changed portion of a lesion not amenable to excisional biopsy be sampled, this qualitative finding may not always be readily apparent nor correlate with the histologically most advanced area of the neoplasm [10, 12].

In a study of 525 dermatopathology specimens of suspected melanocytic neoplasms, only 192 (37%) were excisional biopsies [23]. The diagnostic certainty for invasive melanoma was 95% for excisional biopsy, 82% for deep shave, 77% for punch, and 67% for superficial shave. For melanoma *in situ*, it was 73% for excisional biopsy, 75% for deep shave, 44% for punch, and 42% for superficial shave. The authors emphasized that the goal of biopsy is to identify malignancy versus benignancy and commented that microstaging details are immaterial if the diagnosis is missed. Conversely, a study of 583 melanoma cases not suspected to be melanoma at the time of biopsy found that 16% of shave and 68% of punch biopsies were inadequate for assessment. Punch biopsies greater than 5 mm were diagnostic in 84% of cases [18]. 

Two studies evaluated upstaging of melanoma after diagnostic incisional biopsy with removal of <50% of the surface area of the melanoma. In a series of 46 patients with actinically damaged skin, 40% were upstaged after WLE and 20% had a final diagnosis of invasive melanoma when no invasive melanoma was identified on the biopsy [10]. The authors suggested that limited sampling is inadequate for accurate diagnosis of pigmented lesions on actinically damaged skin. In a second series, 250 patients underwent similar limited incisional biopsy, and 53 patients (21%) were upstaged after definitive excision, including 5 cases where ulceration was identified only after WLE [12]. While the proportion of our patients upstaged after incisional biopsy (24%) is comparable, we did not record the percentage of the lesion biopsied nor did we stratify patients on this basis.

Among studies evaluating the accuracy of various types of diagnostic biopsies for melanoma, one reported that shave biopsy underestimated tumor thickness by 7% and punch biopsy underestimated tumor thickness by 19%, while excisional biopsy was correct in all cases [11]. Decreasing accuracy with increasing melanoma thickness was observed as we noted in our study. In a large Australian series, microstaging inaccuracy was 34% for punch, 19% for shave, and 9% for excisional diagnostic biopsies, but this study compared the unreviewed outside biopsy report to expert dermatopathology review of the final excisional specimen [8]. The investigators reported greater microstaging inaccuracy with increasing tumor thickness, punch or shave biopsy, multiple biopsies, hypomelanotic melanomas, and nonlentigo maligna histology, but not with the proportion of lesion biopsied, anatomic location, or physician specialty. 

Stell and colleagues reported on 223 melanomas diagnosed by excisional biopsy in 23%, punch in 20%, and shave in 57% [13]. Positive deep margins were present in 22% of shave, 7% of punch, and 2% of excisional biopsies. In 161 cases with corresponding WLE data, residual melanoma was identified in 11% after excisional, 20% after shave, and 56% after punch biopsy. The residual tumor was thicker in none after excisional, 4% after shave, and in 26% after punch biopsy, leading to a T-stage change in no cases after excisional, 2% after shave, and 12% after punch biopsy, representing an overall T-stage change in 6 patients (4%). However, the authors noted that cauterization, as is often done after non-excisional biopsies, may destroy or alter residual tumor in the biopsy bed and lead to a false finding of no residual tumor in the WLE specimen. They cautioned that even with negative deep margins, microscopic tumor may be present, but undetected, and that the biopsy may underrepresent true tumor thickness. They stated that while only 2% of their shave biopsy cases were upstaged after WLE, “the inherent difficulty in assigning an accurate Breslow thickness to a previously biopsied tumor” suggested this was actually misleadingly low [13]. Other investigators propose that shave biopsy is best for thinner lesions with a final depth less than 1 mm, a feature difficult to determine from clinical inspection in the absence of obvious signs of advanced disease [11, 14]. 

Two prior studies have examined changes in the recommended surgical management of melanoma patients secondary to discordant histopathology results following WLE. Both studies examined patients diagnosed solely by shave biopsy. Moore and colleagues reported on 139 melanoma patients and found that 54 (39%) had no residual melanoma identified after WLE, 67 (48%) had residual melanoma with a tumor thickness of less than or equal to the biopsy depth, and 18 (13%) had thicker melanoma identified [14]. Of these 18 patients, only 7 (5% of entire series) required additional treatment. Another study of melanomas <2 mm in thickness or melanoma *in situ* reported a positive deep biopsy margin in 37% of patients. After WLE, residual melanoma was identified in 22%, but tumor upstaging occurred in only 3% of patients overall. Of these patients, 89% had a positive deep biopsy margin. Additional surgery for wider margins was recommended for 2% of the patients in the series and performance of a sentinel lymph node biopsy for 1.3%. However, the threshold for sentinel lymph node biopsy was low at a tumor thickness of ≥0.75 mm, likely underestimating the potential change for a recommendation for sentinel node surgery in most melanoma practices where this may not be offered so liberally for thin melanoma patients [15]. Nonetheless, the findings from these two studies closely parallel our results; that is, that 7% of patients diagnosed by shave biopsy were upstaged and 5% required an alteration in treatment recommendations after WLE. To our knowledge, ours is the first study to evaluate changes from a variety of dermatopathologist-reviewed diagnostic biopsy types compared to final histology after WLE.

Our data suggest that subtotal biopsy of melanomas may be inadequate for microstaging, while excisional biopsy provides more accurate and complete information. Overall, we found that only 2% of patients diagnosed by excisional biopsy, but 23% of patients diagnosed by biopsies with positive deep and lateral margins needed additional cancer treatment after final pathology at WLE. We agree that the most important goal is to diagnose melanoma, and that this often can be done with techniques simpler than excisional biopsy, such as deep scoop shave biopsy, potentially decreasing costs and increasing the likelihood of timely diagnosis, prompt treatment, and improved outcomes. 

Limitations of our study include the biases inherent to a retrospective registry study design and the need to exclude some patients in this otherwise consecutive series because of insufficient data for analysis. However, our study is unique in evaluating melanoma patients diagnosed by a variety of biopsy techniques, in comparing biopsy findings after expert dermatopathologist review to the final histopathology from WLE, and in evaluating the effect of any discrepancy on the need for further treatment.

## 5. Conclusions

We found that most diagnostic biopsies were margin positive regardless of biopsy technique, and that more than one third of patients had residual melanoma on WLE. However, with dermatopathology review of the diagnostic biopsy material, T-stage changed in only 8% of patients, and treatment recommendations changed for only 6% of patients. The likelihood of such changes varied distinctly with biopsy type and with the extent of biopsy margin involvement. These findings have clinical utility and suggest that dermatopathologist-reviewed excisional biopsy is preferred when feasible. Our data provide valuable information to inform patient discussion regarding the likelihood of a change in prognosis and treatment recommendations, after WLE, based upon the diagnostic biopsy type and biopsy margin status. 

## Figures and Tables

**Table 1:** Demographic and tumor features of 332 cutaneous melanoma patients.

Variable	*n* (%)
Sex	
Female	174/332 (52%)

Age, years	
Mean, median, range	64.5 ± 0.9, 68, 20–97

Primary tumor anatomic site	
Head and neck	52/332 (16%)
Trunk	110/332 (33%)
Extremities	170/332 (51%)

Primary tumor histologic subtype	
Superficial spreading	187/332 (56%)
Lentigo maligna	82/332 (25%)
Nodular	40/332 (12%)
Acral lentiginous and other	23/332 (7%)

T stage primary tumor (final)	
is*	87/332 (26%)
1	141/332 (42%)
2	50/332 (15%)
3	29/332 (9%)
4	25/332 (8%)

Primary tumor thickness, mean, mm**	1.68 ± 0.16

Primary tumor ulceration, present	46/332 (14%)

Stage (final)	
0	87/332 (26%)
1	175/332 (53%)
2	51/332 (15%)
3	19/332 (6%)

*Tis refers to melanoma *in situ*.

**Tumor thickness calculated for patients with a final diagnosis of invasive melanoma, *n* = 245.

**Table 2:** Frequency and extent of positive margins by biopsy type.

	Biopsy type				
	Excisional	Shave	Punch	Incisional	*P* value
Any margin positive	81/187 (43%)	43/60 (72%)	63/68 (93%)	17/17 (100%)	<0.0001
Type of involved margin (s)					<0.0001
Margin not specified	28/187 (15%)	10/60 (17%)	27/68 (40%)	12/17 (71%)	
Lateral margin only	38/187 (20%)	14/60 (23%)	25/68 (37%)	1/17 (6%)	
Deep margin only	15/187 (8%)	4/60 (7%)	1/68 (2%)	1/17 (6%)	
Lateral + deep margins	7/187 (4%)	15/60 (25%)	10/68 (15%)	3/17 (18%)	

**Table 3:** Frequency of residual melanoma on wide local excision by biopsy technique.

Residual melanoma on WLE	Present	Absent	*P* value
Biopsy technique			
Excisional	42/187 (23%)	145/187 (77%)	Reference
Shave	16/60 (27%)	44/60 (73%)	0.5508
Punch	47/68 (69%)	21/68 (31%)	<0.0001
Incisional	16/17 (94%)	1/17 (6%)	<0.0001

WLE: wide local excision.

**Table 4:** Frequency of residual melanoma on wide local excision by biopsy margin status.

Residual melanoma on WLE	Present	Absent	*P* value
Biopsy margin status			
Negative	14/128 (11%)	114/128 (89%)	Reference
Any margin positive	107/204 (52%)	97/204 (48%)	<0.001
Deep margin positive	25/49 (51%)	24/49 (49%)	<0.001

WLE: wide local excision.

**Table 5:** Association between patient and tumor variables and change in T Stage and the need for additional treatment after wide local excision.

	T Stage change			Treatment change		
	Yes	No	*P* value	Yes	No	*P* value
Age, years, mean	66.0 ± 3.0	64.4 ± 0.9	0.5930	65.6 ± 3.4	64.4 ± 0.9	0.7445

Sex			0.4556			0.6529
Female	16/174 (9%)	158/174 (91%)		12/174 (7%)	162/174 (93%)	
Male	11/158 (7%)	147/158 (93%)		9/158 (6%)	149/158 (94%)	

Primary tumor site			0.5473			0.3707
Head and neck	3/52 (6%)	49/52 (94%)		3/52 (6%)	49/52 (94%)	
Trunk	12/110 (11%)	98/110 (89%)		10/110 (9%)	100/110 (91%)	
Extremities	12/170 (7%)	158/170 (93%)		8/170 (5%)	162/170 (95%)	

Primary tumor histology			0.075			0.1795

Superficial spreading	17/187 (9%)	170/187 (91%)		15/187 (8%)	172/187 (92%)	
Lentigo maligna	2/82 (2%)	80/82 (98%)		2/82 (2%)	80/82 (98%)	
Nodular	5/40 (13%)	35/40 (87%)		3/40 (8%)	37/40 (92%)	
Acral lentiginous and other	3/23 (13%)	20/23 (87%)		1/23 (4%)	22/23 (96%)	

Melanoma diameter, cm	2.25 ± 0.2	1.15 ± 0.1	<0.0001	2.22 ± 0.2	1.17 ± 0.1	<0.0001

Tumor thickness, mm	1.56 ± 0.2	2.6 ± 0.5	0.0401	1.62 ± 0.2	2.29 ± 0.6	0.2538

Biopsy type			<0.0001			<0.0001
Excisional	3/187 (2%)	184/187 (98%)		3/187 (2%)	184/187 (98%)	
Punch	16/68 (24%)	52/68 (76%)		12/68 (18%)	56/68 (82%)	
Shave	4/60 (7%)	56/60 (93%)		3/60 (5%)	57/60 (95%)	
Incisional	4/17 (24%)	13/17 (76%)		3/17 (18%)	14/17 (82%)	

Biopsy margin status			0.002			0.002
Negative	2/128 (2%)	126/128 (98%)		2/128 (2%)	126/128 (98%)	
Positive	25/204 (12%)	179/204 (88%)		19/204 (9%)	185/204 (91%)	

Positive biopsy margin type			0.002			0.02
Positive, not specified	11/77 (14%)	66/77 (86%)		7/77 (9%)	70/77 (91%)	
Positive, lateral only	4/74 (5%)	70/74 (95%)		4/78 (5%)	74/78 (95%)	
Positive, deep only	0/14 (0%)	14/14 (100%)		0/14 (0%)	14/14 (100%)	
Positive, lateral + deep	10/35 (29%)	25/35 (71%)		8/35 (23%)	27/35 (77%)	
